# In vivo multi-parameter mapping of the habenula using MRI

**DOI:** 10.1038/s41598-023-28446-x

**Published:** 2023-03-07

**Authors:** Giorgia Milotta, Isobel Green, Jonathan P. Roiser, Martina F. Callaghan

**Affiliations:** 1 Wellcome Centre for Human Neuroimaging, UCL Queen Square Institute of Neurology, University College London, London, WC1N 3AR UK; 2 Program in Neuroscience, Harvard Medical School, Boston, MA 02115 USA; 3 UCL Institute of Cognitive Neuroscience, 17 Queen Square, London, WC1N 3AZ UK

**Keywords:** Magnetic resonance imaging, Brain imaging

## Abstract

The habenula is a small, epithalamic brain structure situated between the mediodorsal thalamus and the third ventricle. It plays an important role in the reward circuitry of the brain and is implicated in psychiatric conditions, such as depression. The importance of the habenula for human cognition and mental health make it a key structure of interest for neuroimaging studies. However, few studies have characterised the physical properties of the human habenula using magnetic resonance imaging because its challenging visualisation in vivo, primarily due to its subcortical location and small size. To date, microstructural characterization of the habenula has focused on quantitative susceptibility mapping. In this work, we complement this previous characterisation with measures of longitudinal and effective transverse relaxation rates, proton density and magnetisation transfer saturation using a high-resolution quantitative multi-parametric mapping protocol at 3T, in a cohort of 26 healthy participants. The habenula had consistent boundaries across the various parameter maps and was most clearly visualised on the longitudinal relaxation rate maps. We have provided a quantitative multi-parametric characterisation that may be useful for future sequence optimisation to enhance visualisation of the habenula, and additionally provides reference values for future studies investigating pathological differences in habenula microstructure.

## Introduction

The habenula is a small, epithalamic brain structure situated between the mediodorsal thalamus and the third ventricle, rostral to the posterior commissure^1,2^. It is thought to be involved in basic neural mechanisms that are key to survival, in particular aversive processing^3^. It receives inputs from the limbic forebrain via the stria medullaris, and sends efferents to the midbrain via the fasciculus retroflexus, forming a transmission system from forebrain to monoaminergic midbrain known as the dorsal diencephalic conduction system^4^. Given this connectivity, the habenula is uniquely poised to convey—and mediate—the flow of information from the limbic forebrain to key midbrain regions involved in regulating mood and emotional behaviours. It has long been known to be centrally involved in processes such as sleep, aversion, pain, sexual and maternal behaviours, and anxiety. Lesions to and stimulation of the habenula have been shown to alter behaviour in each of these domains^5^.

Interest in the habenula has intensified in recent years due to findings that the lateral habenula (LHb) influences dopaminergic processing of valenced stimuli, i.e. rewards and punishments, and cues indicating their delivery^6^. Dopaminergic neurons in the ventral tegmental area (VTA) and substantia nigra (SN), to which the LHb sends disynaptic inputs, are responsive to rewards and punishments, activating robustly to both rewards and reward-predictive cues, and showing altered activity in response to aversive stimuli and cues^7^. Direct stimulation of the LHb has been shown to profoundly inhibit activation in midbrain dopamine neurons^8,9^, and lesions of the habenula reduce inhibition in the dopaminergic midbrain following reward omission (see Tian and Uchida^10^).

The privileged position the LHb plays in regulating dopamine neuron function has prompted investigation of its role in depression given that several core symptoms, such as anhedonia and disrupted decision-making, have been linked to dopamine dysfunction^11^. The handful of human neuroimaging studies examining habenula activation have verified that it is activated upon delivery of cues predicting aversive outcomes, and inhibited in response to cues predicting reward^12^; and compelling studies of habenula activation in individuals with depression have repeatedly shown altered activation^13–17^. However, the direction and context specificity of these changes remains to be clarified, and future human neuroimaging studies will be essential to clarify the precise nature of altered habenula function in conditions such as depression.

The importance of the habenula for human cognition and mental health thus make it a key structure of interest for neuroimaging studies. However, relatively few studies have been conducted in humans because habenula visualisation in vivo is challenging, primarily due to its subcortical location and small size^18–21^ in the range of 15–30 mm^3^. Studies to date have focused on characterizing the morphology^1,21^, connectivity^2,4,22^ or functional role^14–17^ of the habenula, but few reports have characterized its physical properties using magnetic resonance imaging (MRI)^23–25^ despite these being central to improving its visualisation and understanding its microstructure and functional importance.

Anatomical visualization of the habenula, and robust definition of its boundaries, is contingent on having sufficient image contrast between the habenula and its surroundings. Imaging of the habenula has primarily exploited its high density of myelinated fibres^18,19^, which reduce the longitudinal relaxation time (T_1_) enhancing its visualization on T_1_-weighted images^18^. For this reason, T_1_-weighted images have most commonly been used to manually segment the habenula^18,19^. Schenck et al.^25^ observed increased magnetic susceptibility in the habenula, suggesting an iron-rich sub-structure^26^ as observed for several other sub-cortical brain regions^27,28^. Increased iron content also serves to shorten T_1_ times^29^. Quantitative susceptibility mapping (QSM) has shown promising results in characterizing the origin of contrast, facilitating habenula visualization^24,25^. Nevertheless, habenula delineation and characterization remains challenging due to its small size, which makes it vulnerable to partial volume effects. This motivates a push towards higher (sub-mm) resolution to improve the precision with which the habenula can be delineated. Yoo et al.^23^ combined high-resolution (< 0.8 mm isotropic) imaging of the habenula with QSM to investigate its sub-structure and provided reference QSM values for future in vivo studies. They found an anterior–posterior gradient in susceptibility values that correlated with visual conspicuity scores. However, this pattern was inconsistent across individuals.

To date, microstructural characterization of the habenula has focused on QSM to disentangle paramagnetic iron and diamagnetic myelin contributions. In this work we complement this characterization with measures of the longitudinal and effective transverse relaxation rates [R_1_ (= 1/T_1_) and R_2_* respectively], proton density (PD) and magnetisation transfer saturation (MTsat) using a high-resolution (0.8 mm isotropic) quantitative multi-parametric mapping (MPM) protocol at 3T, in a cohort of 26 healthy participants. These physical properties are sensitive to key biological features such as myelin, iron and water content, and may therefore inform improvements in habenula visualisation and our understanding of its microstructure^30,31^. For each participant, the habenula was manually delineated, its volume computed and the MPM values extracted from this region of interest (ROI). To investigate which metric was best suited to delineating the habenula we also computed the contrast-to-noise ratio (CNR) between the habenula and the surrounding grey matter (GM) tissue. We further investigated the degree of alignment that could be achieved after spatial normalisation to a group template (Montreal Neurological Institute, MNI), which has previously been reported to be poor^19^.

## Results

### Native space characterisation

**Figure 1:**
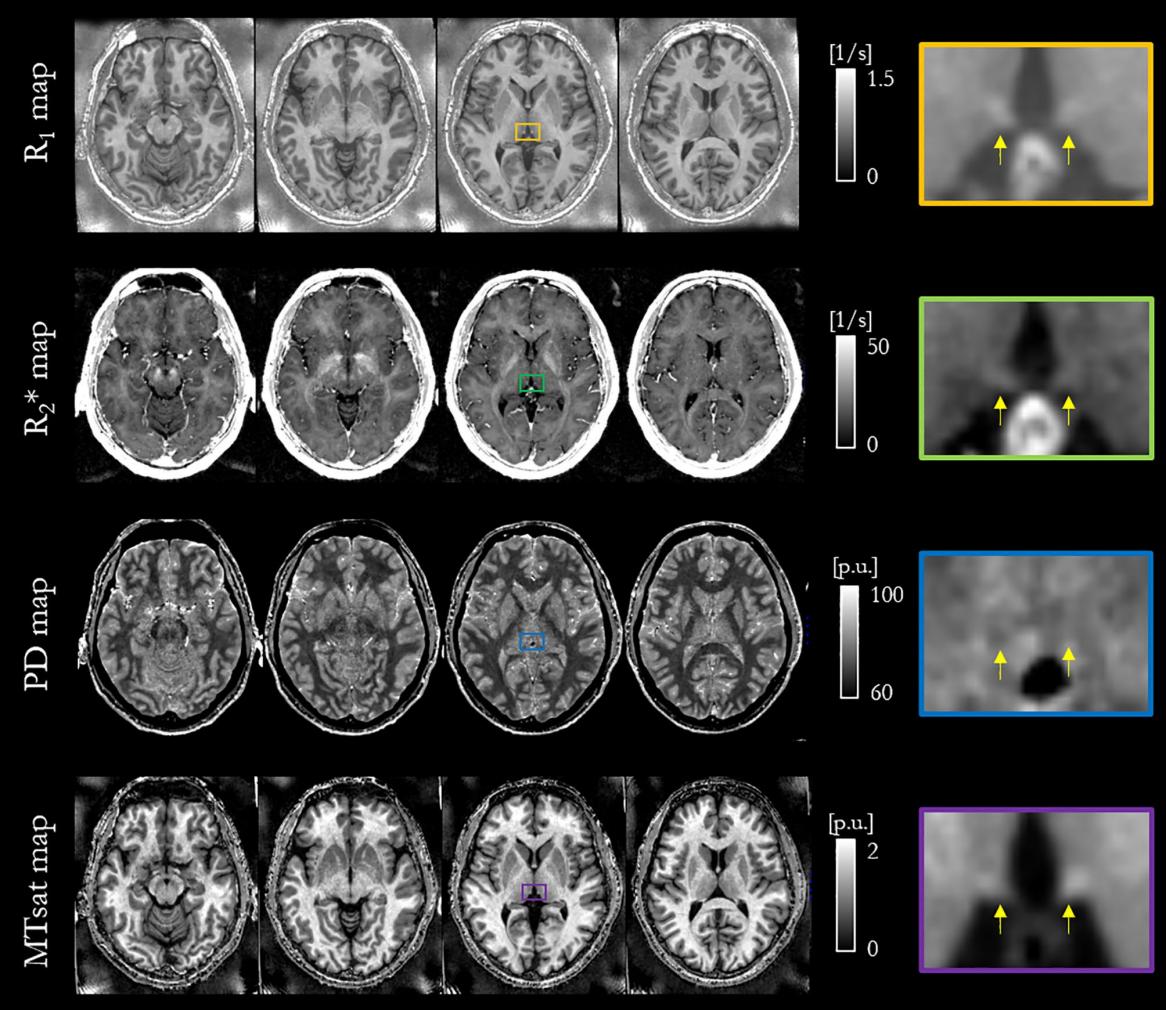
Four transversal planes of R_1_, R_2_*, PD and MTsat maps for one representative participant. On the third transversal plane the left and right habenulae are visible and highlighted by a coloured box, which is enlarged in the final column. Yellow arrows indicate the location of the habenulae on the zoomed view. *P.u.* percent units.

R_1_, R_2_*, PD and MTsat maps were computed for each of the 26 study participants using the hMRI toolbox^32^. MTsat maps from two participants were discarded due to artefacts present in the MT-weighted images. Example maps from one representative participant are shown in Fig. 1 along with zoomed views focusing on the habenula. For a better habenula depiction, Supporting Information Fig. S1 shows the habenula ROI overlaid on the R_1_ map for another representative participant.

**Figure 2:**
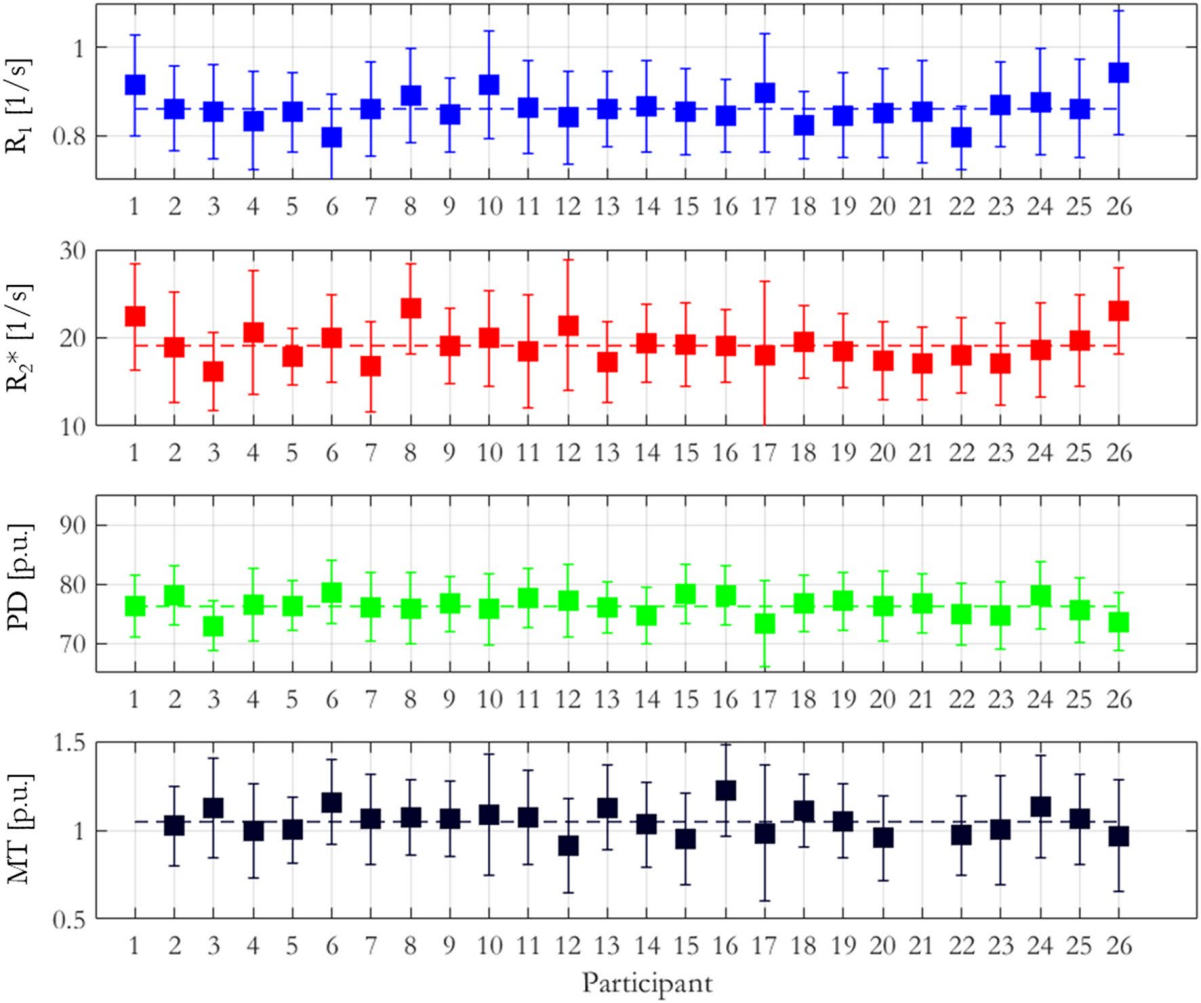
Average R_1_, R_2_*, PD and MTsat values from the manually defined bilateral habenula ROIs for each participant. The bars indicate plus and minus one standard deviation from the mean within the ROIs, the dashed lines represent the mean values for each map across the entire cohort.

The habenula ROI was manually defined according to the geometric protocol described in Lawson et al^19^. The mean (± standard deviation) of each parameter, across participants, within this habenula ROI was R_1_ = 0.86 ± 0.03 1/s, R_2_* = 19.11 ± 1.87 1/s, PD = 76.21 ± 1.52% and MTsat = 1.05 ± 0.08% (Fig. 2).

**Figure 3:**
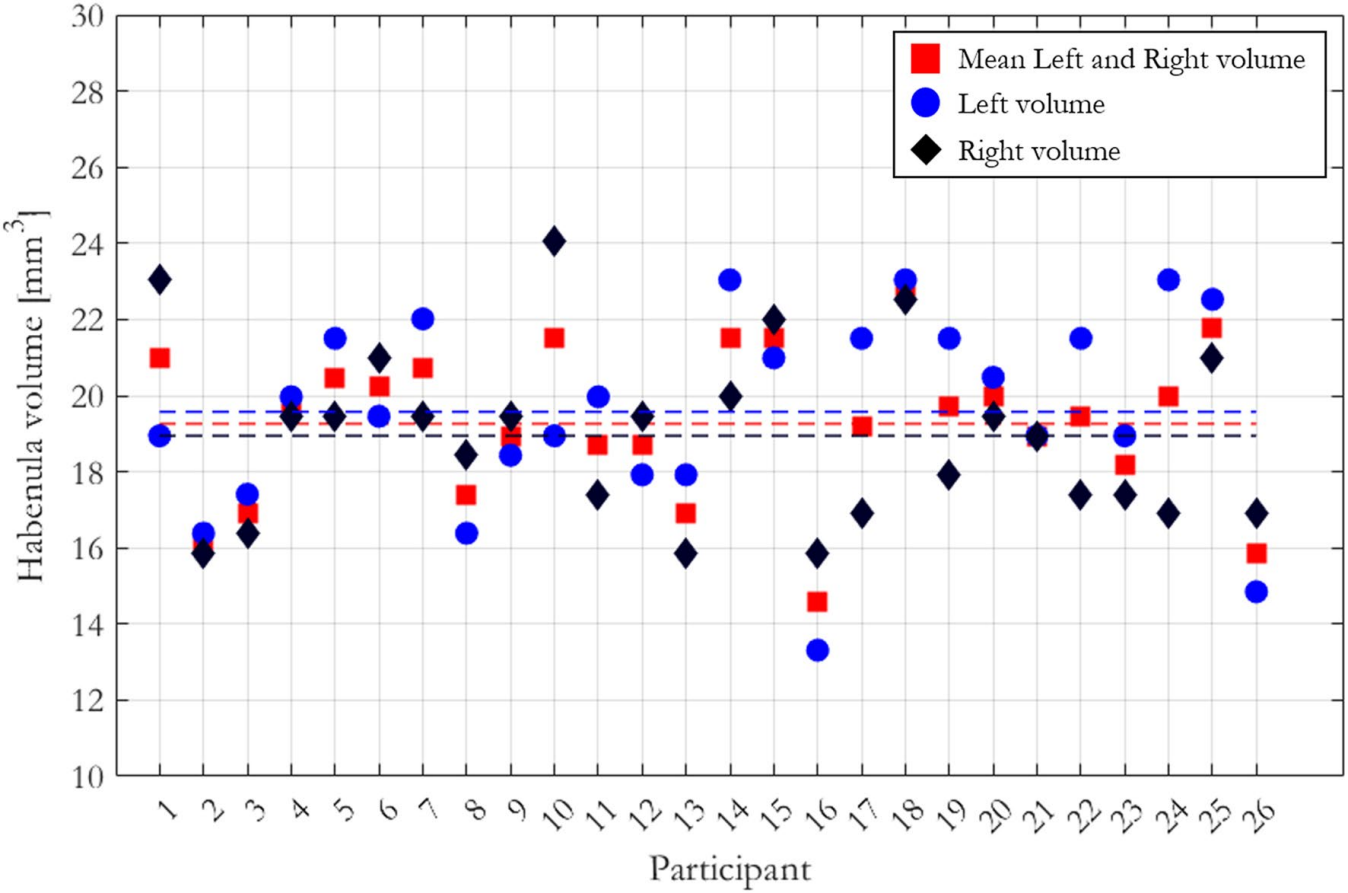
The volume of the left and right habenulae and the mean left and right habenula volumes were computed for each participant. The dashed line represents the mean of these volumes across the cohort.

The left and right habenula volumes and the averaged left and right habenula volume for each participant are shown in Fig. 3. The mean (± standard deviation), across participants, of the averaged volume was 19.26 ± 2.03 mm^3^ with similar left (19.57 ± 2.56 mm^3^) and right (18.94 ± 2.30 mm^3^) habenula volumes. Finally, the mean ± standard deviation of the habenula volume normalised to that of the entire brain, across the cohort, was 8.3 × 10^−3^ ± 1.3 × 10^−3^%.

**Figure 4:**
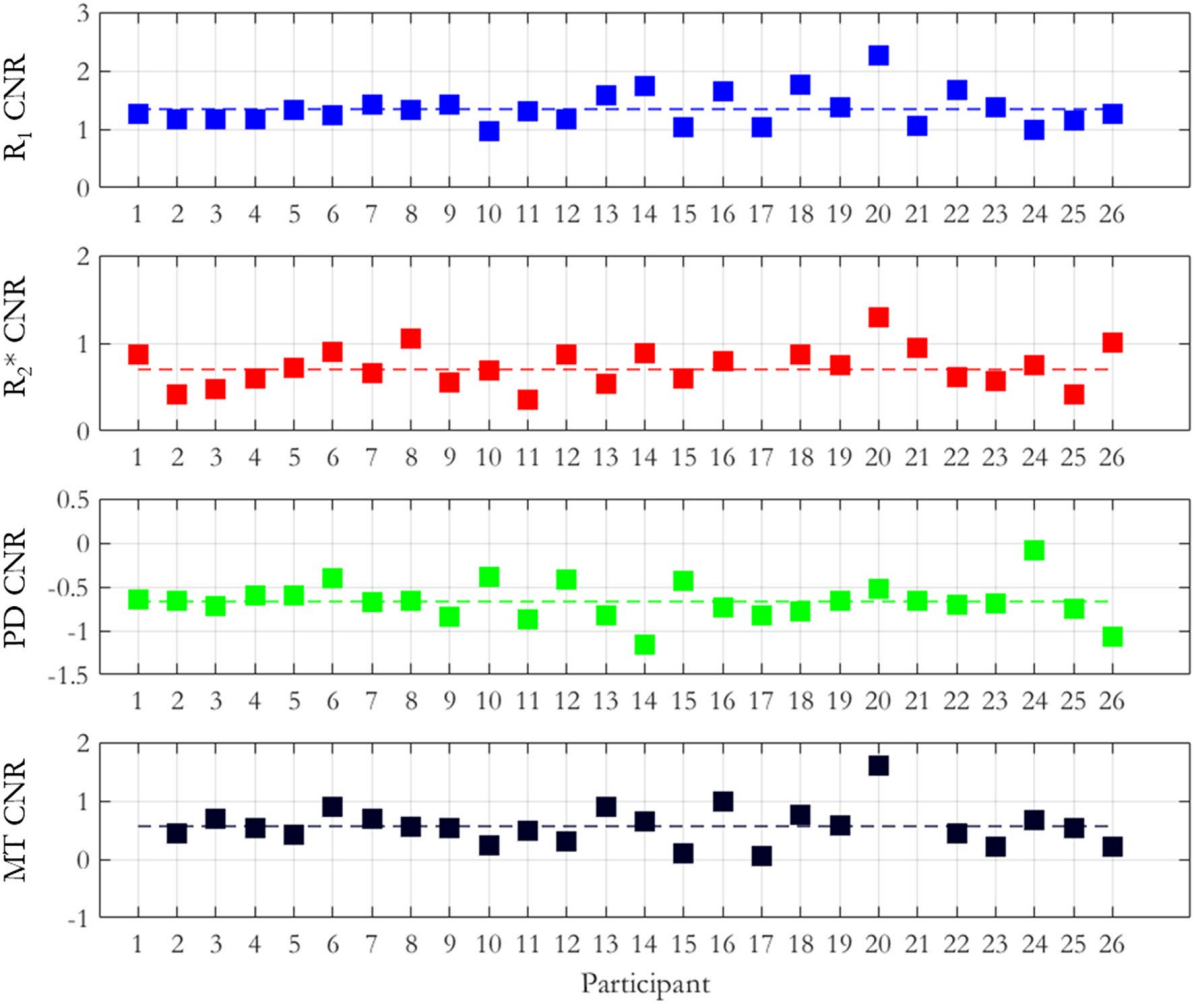
CNR values measured on the R_1_, R_2_*, PD and MTsat maps for each participant. The dashed lines show the mean CNR across the cohort for each map. Note that, because the CNR varies substantially across the maps, different CNR ranges are used to better visualise the variability in CNR across the cohort.

The contrast to noise ratio (CNR) was computed between the habenula and the surrounding grey matter (GM) and is shown in Fig. 4. The R_1_ maps had the highest CNR with a mean (± standard deviation) across participants of 1.34 ± 0.03. The R_2_* and MTsat maps also had positive CNR (i.e. hyperintense habenula relative to the surrounding GM) with mean values across participants of 0.70 ± 0.03 and 0.57 ± 0.33 respectively. On the PD maps, the habenula was hypointense relative to the surrounding GM leading to a negative CNR of − 0.67 ± 0.22.

### Group space CNR

**Figure 5:**
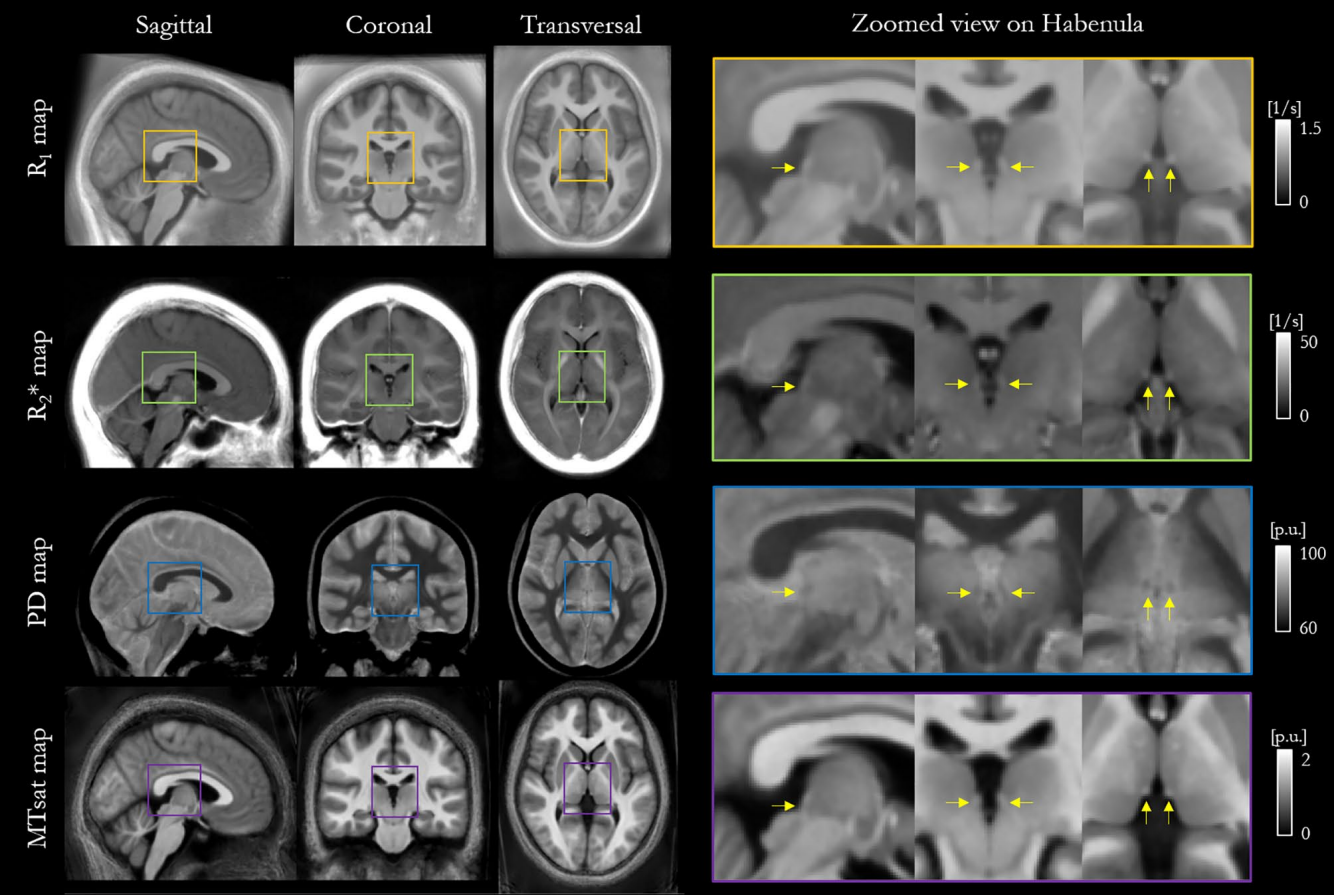
R_1_, R_2_*, PD and MTsat maps in MNI space averaged across the cohort. Sagittal, coronal and transversal views are shown. On the right a zoomed view of the habenulae structures (yellow arrows) are shown for each map.

Spatial normalisation using the Dartel algorithm^33^ was used to align the quantitative maps to normalized space. The cohort-average maps are shown in Fig. 5 along with a zoomed view focusing on the habenula (arrows).

**Figure 6:**
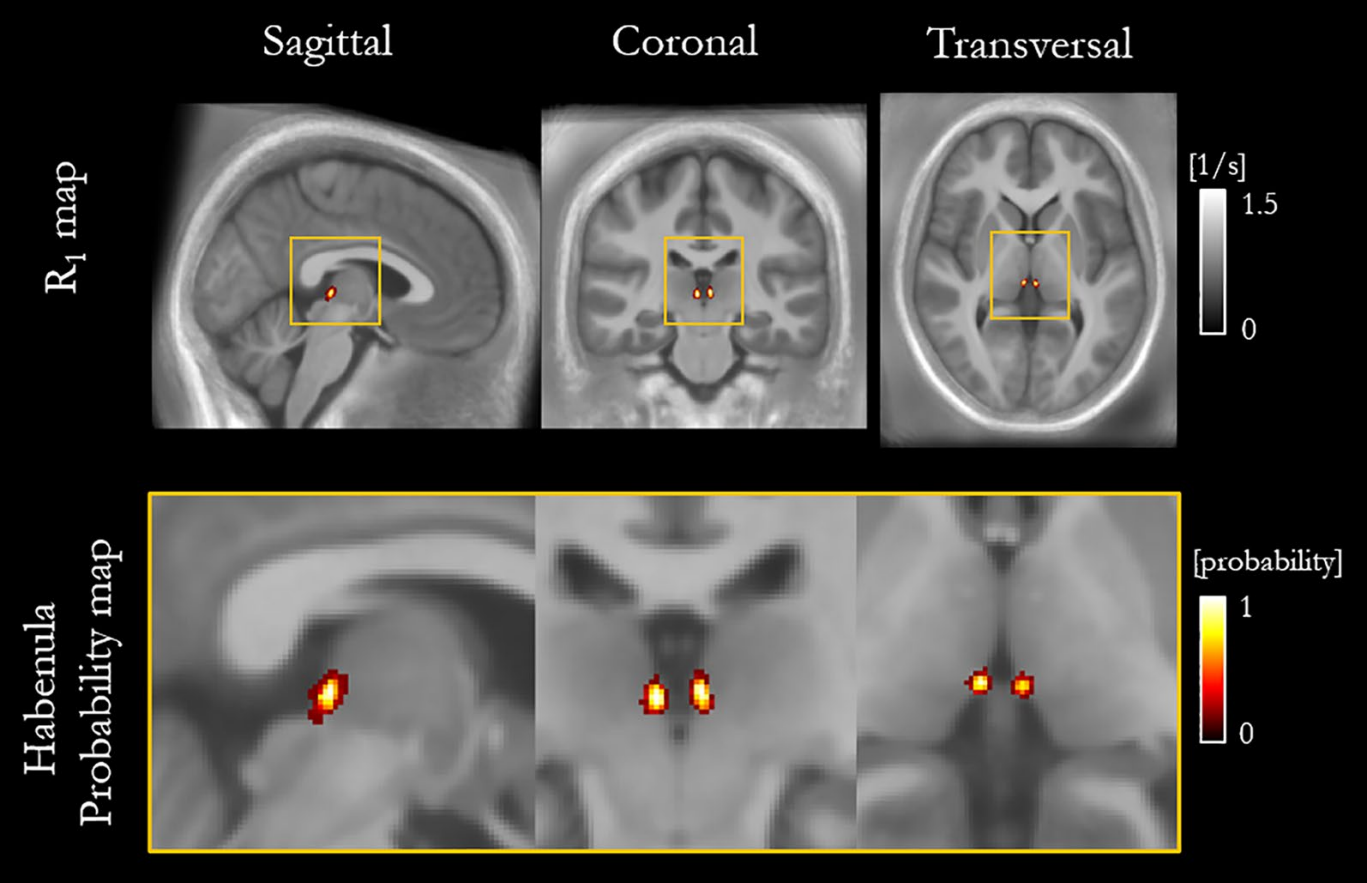
Habenula probability map overlaid on the cohort-average R_1_ map spatially normalised to MNI space (top) and zoomed view of the habenulae (bottom).

Figure 6 indicates the probability, for this cohort, that a voxel lies within the habenula, superimposed on the cohort-average R_1_ map. The maximum probability was 0.77. This amounts to a voxel being consistently defined as being within the habenula for 22 of the 26 participants, but only occurred for two voxels. No voxels were consistently found to be within the habenula for every participant in the cohort.

**Figure 7:**
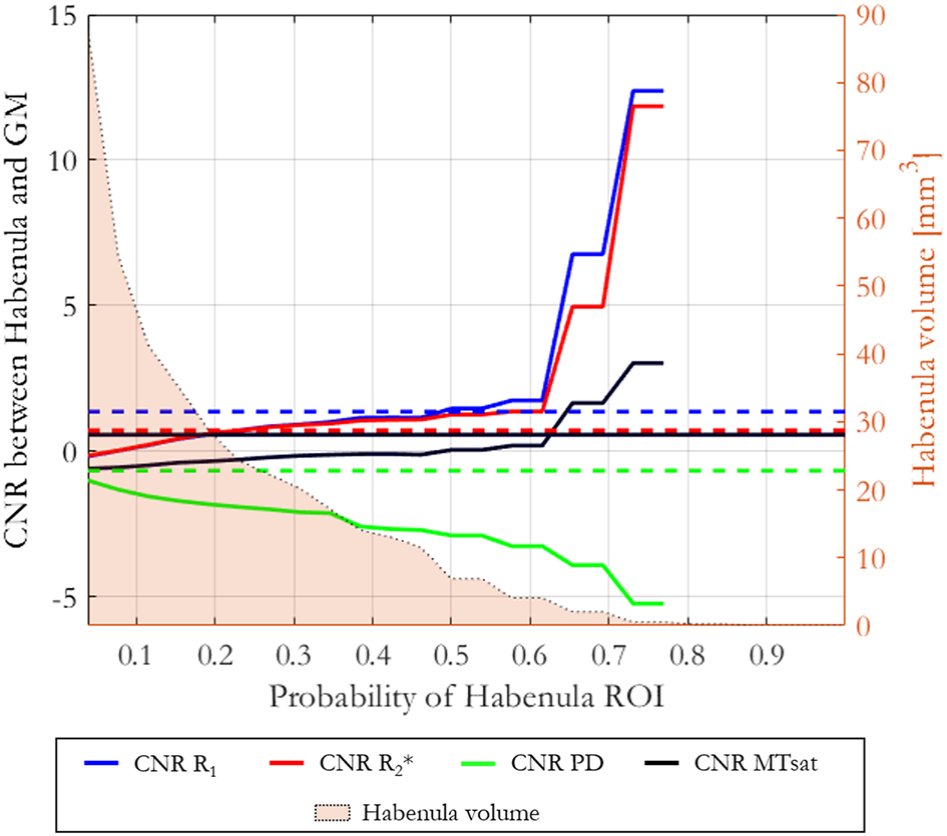
CNR measurements obtained from the R_1_, R_2_*, PD and MTsat maps in normalised space as a function of the probability of voxels being within the habenulae. The filled area represents the average (of right and left) habenula volume in normalised space as a function of the probability threshold used to define the ROIs. For reference, the dashed lines represent the cohort-mean native space CNR.

CNR was also computed on the cohort-average maps in normalized space (Fig. 7). In keeping with the CNR computed per participant, the R_1_ maps had highest CNR. As the definition of the habenula became more conservative (higher probability of being within the habenula) the CNR increased.

## Discussion

The habenula has attracted much interest in neuroscience studies because it plays an important role in the reward circuitry of the brain and is implicated in psychiatric conditions, such as depression^9,10,17^. However, imaging the habenula remains challenging due to its sub-cortical location and small size, with few reports analysing its microstructural composition in vivo^23–25^.

To address this gap in the literature, we performed a multi-parametric characterisation of the microstructure of the habenula by quantifying relaxation rates (R_1_, R_2_*), water content (PD) and a marker of macromolecular content (MTsat), most notably myelin, in a cohort of 26 healthy participants. The measurements were consistent across the cohort (Fig. 2) and could therefore be used to guide future studies optimising the in vivo visualisation of the habenula. The standardised nature of quantitative MRI measurements also affords the opportunity to compare measurements across different cohorts, imaging sites and time points^34^. As such, these data provide a quantitative baseline from a healthy cohort against which to assess pathological differences.

The habenula was most clearly visualised on the R_1_ map (Fig. 1). Its boundaries were consistent across the different parameter maps. This qualitative observation motivated the use of this map for the manual delineation of the habenula. Subsequent quantitative CNR analyses (Fig. 4) confirmed that the contrast between the habenula and the surrounding GM was consistently highest on the R_1_ maps for each participant. This suggests that, among the four quantitative maps considered in this analysis, the R_1_ map is the most suitable choice for segmenting the habenula. In prior habenula research T1-weighted images have been most commonly used for this purpose. R_1_ maps mirror the contrast in T1-weighted images, but additionally offer the advantage of not being confounded by hardware-driven contrast (e.g. due to spatial variation in transmit and receive fields) and of having less vulnerability to other sequence settings (e.g. the echo time dictates the degree of additional T_2_* weighting in a T_1_-weighted image but is corrected for in an R_1_ map). Therefore, R_1_ maps might also be preferable for more reliable habenula segmentation with respect to T1-weighted images.

The habenula was segmented on R_1_ maps, whereas the segmentation procedure described in^19^ (which was followed for the delineation of the ROIs) was performed on T_1_-weighted images. In this work, a lower mean volume across the left and right habenulae was observed in comparison to reports in the literature using MRI^19,23^ or post mortem histology^20^. In vivo, the difference in resolution and image contrast^35–37^ is likely to have an impact on the segmentation procedure and therefore the results of the volumetric analysis. For example, Lorio et al.^36^ demonstrated that age-related differences in iron content can lead to differential image contrast depending on the sensitivity to signal decay (i.e. R_2_* effects) and therefore to variable estimates of atrophy. Being such a small structure, the surface area of the habenula is proportionately high making it particularly vulnerable to partial volume effects and the resolution of the data used to define its boundary. Although our volume measurements were lower than previously reported values using MRI^19^ or histology^20^, the precision of our measurements was higher (SD of 2.03 mm^3^ here vs. 3.7 mm^3^ reported in^19^ and approximately 5.2 mm^3^ post mortem) and were consistent between left and right habenulae, suggesting robust delineation across the cohort. In the in vivo context, this gain in precision may also be driven by the use of higher resolution data.

The means, across the cohort, of the spatially normalised R_1_, R_2_*, PD and MTsat maps (Fig. 5) show a reduction in noise, enabling the visualisation of the habenula in every map. In particular, the habenula is visible on the normalized PD map, whereas, on the per-participant PD maps, it was obscured by noise. Despite the high contrast observed in normalised space, the overlap of the habenula across the cohort was poor as evidenced by the probability map. A maximum probability of 0.77 was obtained, meaning that across the cohort (N = 26 total) only 22 participants had a voxel commonly attributed to being within the habenula. Furthermore, this maximum probability occurred for only two voxels in the habenula. The fact that very few voxels were coincident across the cohort indicates that the spatial normalisation algorithms available today cannot adequately align this small structure^38^. This finding speaks against the use of probabilistic maps being used as an atlas to define the habenula for subsequent studies. However, it could be used as a template to approximately locate the habenula, e.g. to provide a seed location to initialize automatic segmentation routines^18^. Such automated segmentation routines may also benefit from using one, or some optimal combination, of the quantitative maps as input.

The CNR analysis on the normalized maps reflected what was observed on a per-participant basis (dashed lines in Fig. 7) only when the probability threshold used to define the habenula ROI exceeded 0.3. As shown in Fig. 7, the volume of the habenula defined by thresholding the ROI with this probability is 20.2 mm^3^, which approximately matches the mean volume measured across the cohort in native space (Fig. 3). If a broader habenula ROI is defined (threshold < 0.3), voxels belonging to GM and ventricular cerebrospinal fluid (CSF) are included in the ROI (as indicated by the overestimated habenula ROI volume) biasing the CNR measurements. When a stricter definition of the habenula is adopted (threshold > 0.3) voxels with partial volume between the habenula and surrounding GM or CSF are excluded from the analysis and thus an increase in CNR is observed. The noise reduction obtained due to averaging following spatial normalization further improves the CNR. Consistent with native space findings, the highest CNR is obtained on the normalized R_1_ maps.

One of the main limitations of the proposed work is that the habenula ROIs were defined by a single individual (IG), and no reliability analysis was performed. However, the delineation of the habenulae followed a well-defined procedure^19^ and the capacity to visualise the habenula was bolstered by the use of an R_1_ map with high spatial resolution. The cohort size was also modest. However, as noted previously, the consistency of the measurements bodes well for future optimisation of habenula visualisation. However, a larger cohort would be needed to confirm this consistency in microstructure or to assess any meaningful inter-individual variation^39^. CNR was only measured between habenula and GM. The contrast between the habenula and ventricular CSF was not considered in the analysis. This approach was adopted because the contrast between the CSF and habenula is usually sufficiently high to aid its visualization, whereas delineation with respect to GM is usually more challenging. However, the contrast with respect to CSF is particularly high on the MTsat map, but this was not captured in the CNR analysis due to its focus on GM.

With suitable computation of the signal phase, the MPM protocol could naturally be augmented to include QSM without any extension of the acquisition time. Given that QSM has shown promise in depicting the habenula, suggesting the presence of iron-rich substructure, this would provide a more complete characterisation of habenula microstructure.

## Conclusion

We have provided a quantitative multi-parametric characterization of the habenula microstructure in vivo in a sample of 26 healthy participants. The results suggest that the habenula can be most clearly visualised on longitudinal relaxation rate (R_1_) maps. This characterisation provides baseline measurements that may be useful for sequence optimization to further enhance visualisation, and provide reference values for future studies investigating pathological differences in habenula microstructure.

## Methods

### Data acquisition

#### MPM protocol

Data were acquired on a Siemens 3T Prisma using a 64-channel head and neck receiver coil. A Multi-Parameter Mapping (MPM) protocol, as described by Callaghan et al.^40^, was used for image acquisition. In brief, it comprised three RF- and gradient-spoiled multi-echo 3D FLASH scans acquired with T_1_ (α_T1w_ = 21°), PD (α_PDw_ = 6°) or MT (α_MTw_ = 6°) weighting. To achieve MT-weighting a 4 ms Gaussian RF pulse at 2 kHz off-resonance with flip angle of 220˚ was applied prior to excitation. The number of acquired echoes for the MT-weighted acquisition was reduced from eight to six in order to maintain a consistent TR of 25.0 ms across all FLASH scans. Gradient recalled echoes were acquired with echo times ranging between 2.30 and 13.87 ms in steps of 2.30 ms. Additional imaging parameters included: isotropic resolution of 0.8 mm, FOV = 256 (HF) × 240 (AP) × 179 (RL) mm^3^, bandwidth of 488 Hz/pixel and a GRAPPA acceleration factor of 2 in each phase-encoding direction, with 40 integrated calibration lines. RF spoiling with an increment of 137° was used in combination with a spoiler gradient moment of 6π every TR, imperfect spoiling was corrected via the hMRI toolbox^41^. To account for field inhomogeneity an effective transmit field (${{B}_{1}^{+}}_{eff}$) map was acquired prior to the acquisition of the 3D FLASH scans as described by Lutti et al.^42^. To correct for inhomogeneity in the receiver field, two additional low-resolution volumes were acquired, with the array and body coils respectively, immediately prior to each of the three weighted FLASH acquisitions. The ratio of these low-resolution (8 mm^3^ isotropic resolution) images was used to estimate the position-specific net receiver sensitivity of the array coil, which was then used to correct for movement between the acquisitions^43^.

#### Imaging sessions

Data were acquired on 26 participants (12 males). The participants were recruited for a functional MRI study. Participants ranged in age from 20 to 43, mean age was 24.36 (standard deviation 4.88), all participant were right handed. All data acquisition was approved by the Central London Research Ethics Committee 3, and all methods were performed in accordance with specified regulations. All participants gave written informed consent for the study prior to data acquisition.

### Data analysis

All images were analysed using SPM12^44^ (Wellcome Centre for Human Neuroimaging, London, UK). R_1_, R_2_*, PD and MTsat maps were generated for each participant using the hMRI toolbox^32^.

The habenula delineation was performed on the R_1_ maps because of its optimal depiction of the habenula. The definition procedure was performed following the geometric approach described by Lawson et al.^19^. R_1_ maps of each participant were rotated and translated until the midpoint of the posterior commissure was aligned with the midpoint of the anterior commissure. On the re-oriented R_1_ maps, the left and right habenulae regions of interest (ROIs) were manually drawn on the re-oriented R_1_ maps. An inverse transformation, including rotation and translation, was then applied to the ROI masks to re-orient them back to the native space of the R_1_ maps.

For each participant the mean and standard deviation of R_1_, R_2_*, PD and MTsat was computed within the habenulae ROI (including both the left and right habenulae). The volume of the left and right habenulae was also computed, along with the average of these, for each participant. Finally, the normalised habenula volume was computed for each participant by dividing the total habenula volume (left and right habenulae combined) by a participant-specific whole brain mask defined as those voxels with a WM, GM or CSF probability greater than 0.9.

Participant-specific grey matter (GM) masks including GM surrounding both the left and right habenula were defined by: (1) extending the habenula ROIs with a spherical augmentation of one voxel, (2) removing the habenula ROIs from the extended mask and (3) combining the mask via logical conjunction with a whole-brain GM mask defined by voxels with a probability greater than 0.9 of belonging to a GM tissue class following segmentation using the unified segmentation algorithm^45^.

The CNR between the habenula and the GM ROI was calculated for R_1_, R_2_*, PD and MTsat maps as:

$$CNR=\frac{mean(Habenulae\; ROI)-mean(GM ROI)}{std(Habenulae\; ROI)}$$

Spatial normalization of the R_1_, R_2_*, PD, MTsat, GM probability maps and binarised habenulae ROI was performed using the Dartel toolbox in SPM. This procedure included the following steps:

1. Generation of GM and white matter (WM) tissues classes via segmentation of the T1-weighted image for each participant.
2. Computation of non-linear deformations to align each of the participants to a common group space, using the GM and WM tissue classes obtained from step 1 to drive the deformation estimation using the Dartel algorithm^33^.
3. Application of the participant-specific deformations from step 2 to the R_1_, R_2_*, PD, MTsat maps, the GM tissue probability maps and the habenula ROIs using a smoothing kernel of 0.8mm^3^.
4. Averaging the maps across the cohort in normalised space.
5. Computation of a habenula ROI probability map.

The CNR between the habenula and surrounding GM defined in normalized space was calculated on the cohort-averaged R_1_, R_2_*, PD and MTsat maps in normalized space. The CNR was measured while varying the probability map threshold defining the habenula. As the probability of being within the habenulae increased, the number of voxels within the habenulae ROI decreased, excluding more voxels that may contain partial volume of the surrounding GM from the analysis. The GM ROI was kept constant and defined by the logical intersection between the average whole-brain GM mask in normalized space and the outer habenula mask. The latter was obtained by extending the normalised and non-thresholded habenula ROIs with spherical augmentation of one voxel and by removing the normalized habenula ROIs.

## Supplementary Information

Supplementary Figure S1.

## Data Availability

The datasets generated and/or analysed during the current study are not publicly available due to ethical and data protection restrictions, but are available from Jonathan Roiser on reasonable request and subject to an institutional data sharing agreement.
