# Supplementary material for: In vivo multi-parameter mapping of the habenula using MRI
Source: Sci Rep. 2023 Mar 7;13:3754. doi: 10.1038/s41598-023-28446-x (PMC9992523; doi:10.1038/s41598-023-28446-x)
Supplement: Supplementary file 1 — Supplementary Figure S1. [file 41598_2023_28446_MOESM1_ESM.pdf]

Supplementary Information Figure

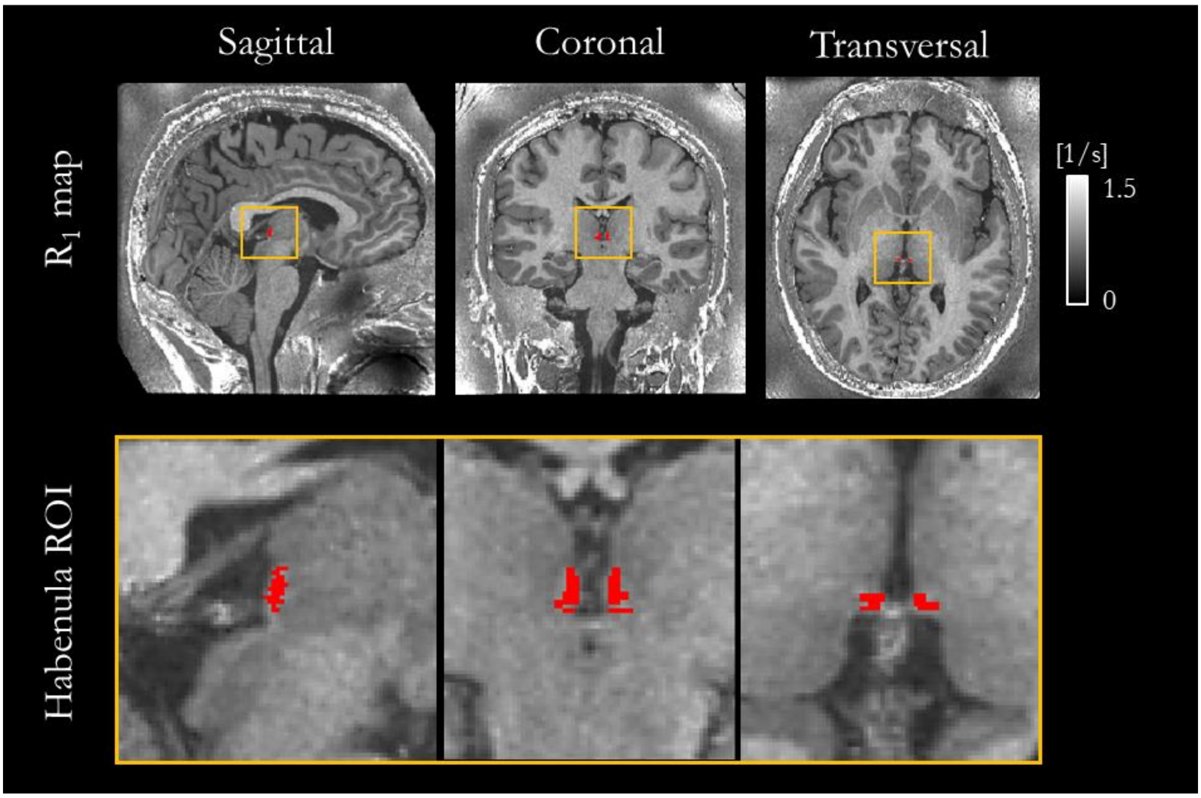

Supporting Information Figure S1 – Habenula ROI overlaid on the R_1 map in native space for one representative participant. A zoomed view of the habenula ROI is shown in the bottom row.
